# The mycobacterial ATP burst is a lysis artifact and serves as an assay for drug-induced cell wall damage

**DOI:** 10.1016/j.celrep.2026.117286

**Published:** 2026-04-22

**Authors:** Claire V. Mulholland, Bei Shi Lee, Alexander Chong, Jinhua Cui, Kevin Pethe, Michael Berney

**Affiliations:** 1 Department of Microbiology and Immunology, Albert Einstein College of Medicine, Bronx, NY 10461, USA; 2 Lee Kong Chian School of Medicine, Nanyang Technological University, Singapore, Singapore; 3 Department of Public and Global Health, EBPI, University of Zurich, Zurich, Switzerland; 4 Singapore Centre for Environmental Life Sciences Engineering, Nanyang Technological University, Singapore, Singapore; 5 National Center for Infectious Diseases (NCID), Singapore, Singapore; 6 Lead contact

## Abstract

Antibiotics targeting the mycobacterial cell wall are a cornerstone of tuberculosis treatment, yet how these drugs facilitate bacterial killing remains incompletely understood. Studies using the BacTiter-Glo luminescence assay have reported increased mycobacterial ATP levels following treatment with cell wall inhibitors such as isoniazid, a phenomenon referred to as an “ATP burst.” This is proposed to contribute to drug-induced killing. Here, we show the ATP burst is not a biological response but rather an experimental artifact resulting from enhanced cell lysis induced by cell-wall-targeting drugs. Mechanical lysis by bead beating abolishes the ATP burst, enabling more reliable assessment of ATP levels. We demonstrate the utility of this approach as a functional readout for identifying compounds that disrupt the mycobacterial cell wall and for screening synergistic or antagonistic interactions with cell wall inhibitors. These findings clarify the mechanistic basis of the ATP burst and provide a practical tool for antimycobacterial drug discovery.

## In brief

Mulholland et al. show that the apparent ATP increase detected by the BacTiter-Glo assay in mycobacteria treated with cell wall inhibitors is a lysis artifact rather than a biological response. This study corrects a common misinterpretation and reframes the assay as a rapid screen for mycobacterial cell wall disruption.

## Graphical abstract

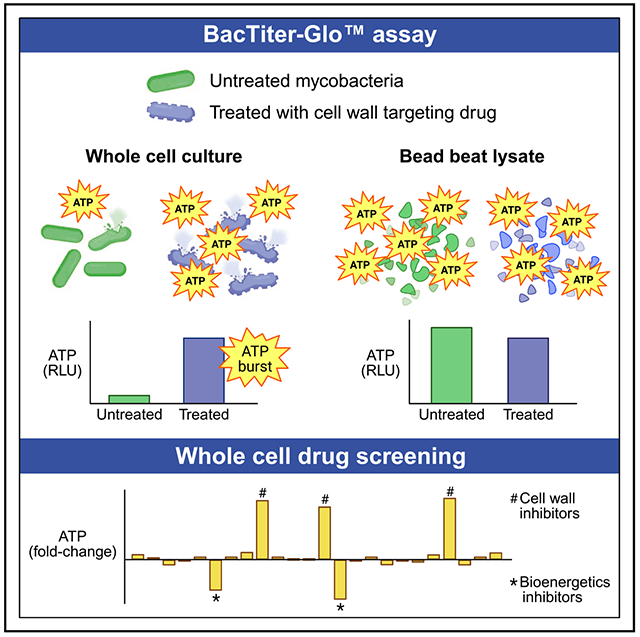

## INTRODUCTION

*Mycobacterium tuberculosis* (*Mtb*) remains one of the world’s deadliest pathogens, causing more than one million deaths annually.^1^ In parallel, infections with non-tuberculous mycobacteria (NTM) have emerged as an escalating public health concern.^2^ In response to the urgent need for therapeutic strategies against mycobacterial diseases, the past three decades have witnessed a marked expansion in molecular research aimed at elucidating the biology of these pathogens. A key aspect of this research has been the measurement of cellular ATP levels as a proxy for metabolic and bioenergetic state, for example, in studies of mycobacterial physiology and drug mechanisms of action,^3–7^ and high-throughput drug screening.^8–10^ Over the past two decades, luciferase-based assay kits, such as BacTiter-Glo, have been widely adopted in mycobacterial research due to their affordability, rapid turnaround time, and ease of use. Notably, a search of PubMed Central for “Bac-Titer-Glo & Mycobacterium” yields over 300 results, reflecting its widespread use. Recent studies using this assay have revealed an intriguing and unexpected phenomenon: treatment of different mycobacterial species (*Mtb*, *Mycobacterium bovis* BCG, *Mycobacteroides abscessus*, and *Mycobacterium smegmatis*) with cell wall biosynthesis inhibitors leads to an apparent increase in ATP levels.^3,4,11–17^ This transient increase, described as an “ATP burst,” has been hypothesized to represent a lethal cellular process^4^ and to implicate electron transport chain perturbation in isoniazid-induced killing.^3^ However, it is still mechanistically unclear if this surge in ATP results from increased ATP production, decreased ATP hydrolysis, or an unknown mechanism, and how this might contribute to bacterial cell death.

Since ATP measurements based on the BacTiter-Glo method have become a standard in mycobacteriology, we sought to elucidate the mechanistic basis of the ATP burst phenomenon. Furthermore, the mycobacterial cell wall is a favorable target space for drug discovery,^18^ and cell wall inhibitors are central components of the multidrug treatments used to combat *Mtb* and NTM infections.^19,20^ Disruption of the mycobacterial cell wall can also improve the uptake and efficacy of other drugs.^21–26^ Conversely, drugs that target bioenergetics reduce the bactericidal efficacy of cell-wall-targeting compounds.^3,4,14,16^ Thus, a better understanding of the mechanisms by which cell wall inhibitors lead to cell death is required to improve combination therapies and may reveal potential new drug targets.

In this work, we show that the ATP burst observed in the BacTiter-Glo assay is an experimental artifact resulting from synergistic lysis between cell-wall-targeting drugs and the lysis buffer of the assay, leading to enhanced ATP release from *Mtb* and other mycobacteria. We demonstrate how this phenomenon can be exploited to identify compounds that target the cell wall and to detect potential synergistic and antagonistic interactions, providing a simple and rapid method to assess drug effects on the mycobacterial cell wall.

## RESULTS

### The ATP burst is an experimental artifact caused by improved lysis following treatment with cell wall inhibitors

Mycobacteria have a thick and complex cell wall that is inherently resistant to lysis by conventional methods. The BacTiter-Glo assay relies on proprietary reagents to induce bacterial lysis; however, commercial systems are generally much less efficient at lysing mycobacteria compared to mechanical methods such as bead beating or heat inactivation.^27–29^ In our preliminary work, we detected no ATP burst in response to isoniazid when *Mtb* cells were heat-inactivated prior to the BacTiter-Glo assay (Figure S1). Since heat inactivation leads to cell lysis, we hypothesized that the ATP burst might be related to lysis rather than an actual increase in cellular ATP levels. To test this systematically, we treated *Mtb* mc^2^6230^30^ with the cell wall inhibitors isoniazid and ethambutol, as well as the ATP synthesis inhibitor bedaqui-line. ATP was then measured with the BacTiter-Glo assay using three different lysis methods: (1) “whole-cell”: incubating whole-cell culture with the BacTiter-Glo reagent for kit-driven lysis, (2) “beat”: bead-beating whole-cell culture followed by a brief heat denaturation, or (3) “heat”: boiling whole-cell culture for 30 min at 100°C. The lysate from both the heat and beat methods was spun down and then used in the BacTiter-Glo assay in the same manner as the whole-cell culture. Cells at high density (OD_600_ 0.33; ~1 × 10^8^ CFU/mL) were treated at 50 × the minimum inhibitory concentration (MIC) to elicit robust responses and provide sufficient biomass for mass spectrometry analysis.

**Figure 1.**
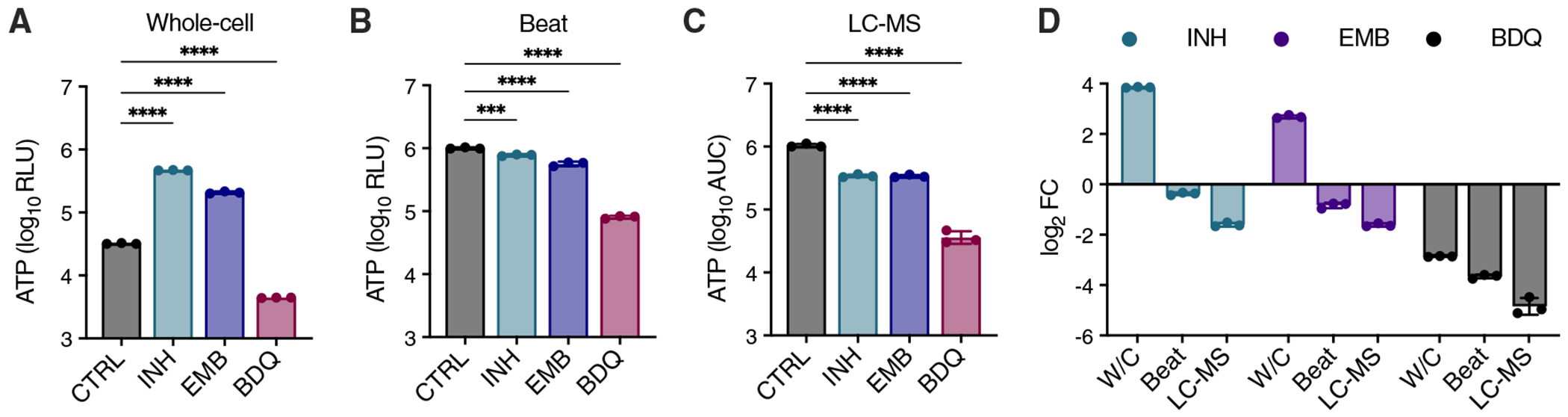
The ATP burst is an experimental artifact *Mtb* mc^2^6230 at OD_600_ 0.33 was treated with isoniazid (INH, 3 μg/mL), ethambutol (EMB, 50 μg/mL), or bedaquiline (BDQ, 12.5 μg/mL) alongside untreated controls for 24 h (~50 × MIC). (A and B) ATP was measured using the BacTiter-Glo assay from either whole-cell broth (‘W/C’) (A) or cells lysed by bead beating (‘Beat’) (B). (C) Intracellular ATP levels in the same cultures measured by LC-MS. (D) Fold change in ATP levels compared to untreated controls for the different methods shown in (A–C). Mean ± SD, *n* = 3 replicate cultures. ***p < 0.001 and ****p < 0.0001 by one-way ANOVA with šídák’s multiple comparison test (MCT). See also Figure S2.

In agreement with previous reports,^3,4,11,13,14^ we observed a significant increase in the ATP signal for isoniazid and ethambutol treatment in the whole-cell assay (14- and 6-fold increase, respectively) (Figure 1A). However, when cells were lysed by bead beating, no increase in ATP was observed, and instead, ATP levels were slightly lower compared to the untreated control (Figure 1B). Concurrently, heat-inactivated samples also failed to show an ATP burst (Figure S2B). Importantly, bead beating of untreated cells increased the ATP signal by approximately 30-fold compared with the whole-cell assay (Figures 1A and 1B), indicating ineffective lysis of mycobacterial cells by the BacTiter-Glo kit. We also tested whether isoniazid or ethambutol alone induced ATP release; however, neither drug substantially increased supernatant ATP (the signal was ≤2% of that observed in the whole-cell assay) (Figure S2E). This indicates that the ATP burst is not a direct consequence of drug-induced lysis, but rather results from cell wall weakening, which enables effective lysis upon exposure to the BacTiter-Glo lysis buffer.

We next sought to confirm relative ATP levels using an independent method and turned to liquid chromatography-mass spectrometry (LC-MS). ATP was extracted into water/acetonitrile/methanol buffer and measured on an Agilent 6545 Q-TOF mass spectrometer. No ATP burst was detected by this method (Figures 1C and S3). Instead, ATP levels were significantly lower in isoniazid- and ethambutol-treated cultures compared to the untreated control. As expected, all methods detected a significant reduction in ATP in cells treated with the ATP synthase inhibitor bedaquiline (Figures 1 and S2).

**Figure 2.**
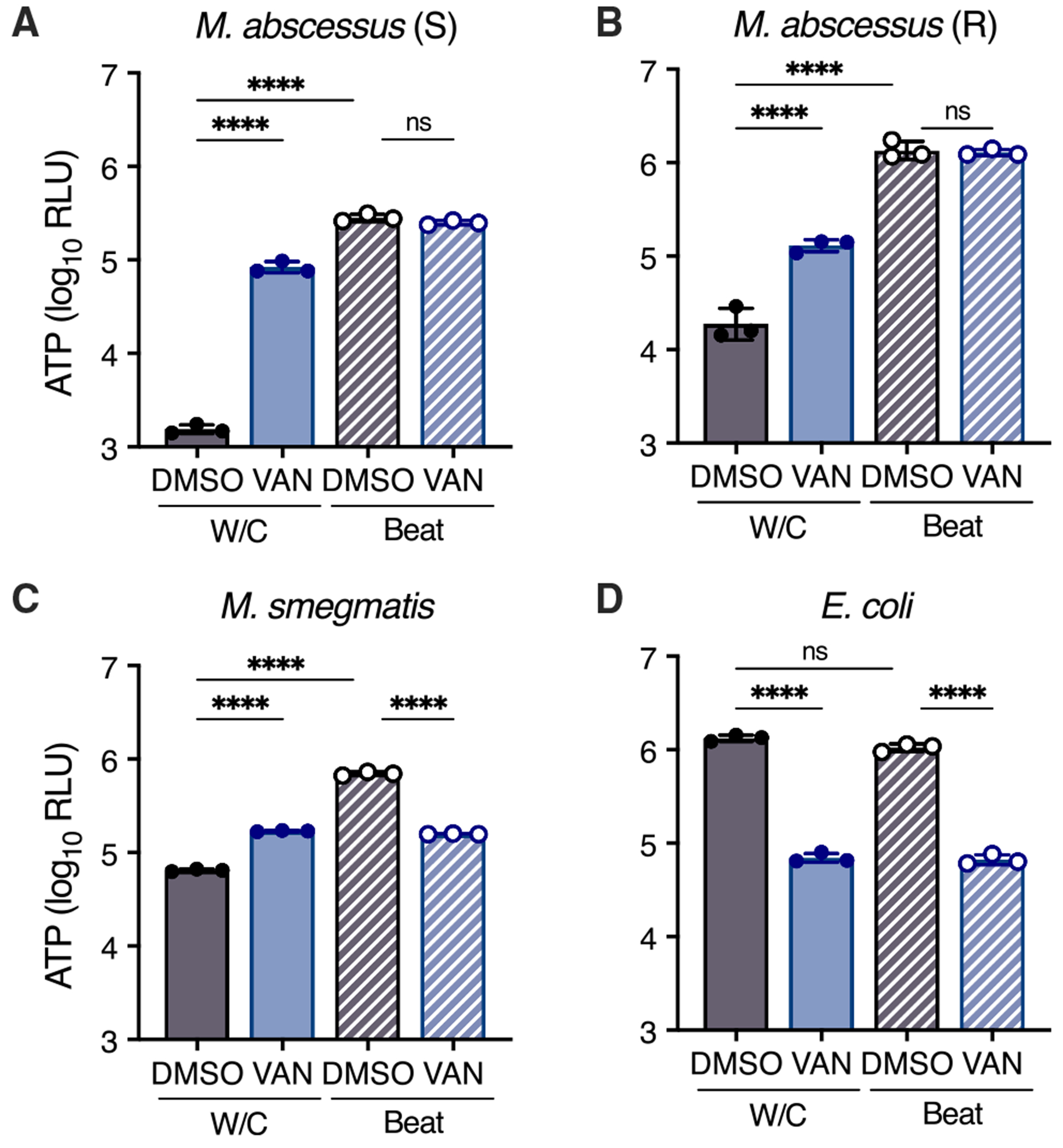
ATP release in the BacTiter-Glo whole-cell assay is impaired in mycobacteria due to the complex cell wall (A–C) *M. abscessus* smooth (A) and rough (B) morphotypes and *M. smegmatis* (C) were treated with 250 μg/mL vancomycin for 4 h. (D) *E. coli* was treated with 500 μg/mL vancomycin for 2 h. ATP was measured using the BacTiter-Glo whole-cell and beat assays. All cultures were treated at OD_600_ 0.033 alongside DMSO-only controls. Mean ± SD, *n* = 3 replicate cultures. ****p < 0.0001 by one-way ANOVA with šídák’s MCT. These experiments were repeated with similar results.

To test if our findings also apply to other mycobacteria, we treated *M. abscessus* and *M. smegmatis*, two non-tuberculous mycobacteria, as well as the Gram-negative bacterium *Escherichia coli*, with the peptidoglycan biosynthesis inhibitor vancomycin at approximately 4–6 × their respective MICs^31–33^ and measured ATP using the BacTiter-Glo whole-cell and beat assays. In agreement with our findings in *Mtb*, vancomycin treatment of *M. smegmatis* and *M. abscessus* significantly increased ATP in the whole-cell assay but not when cells were lysed by bead beating. Furthermore, bead beating increased the ATP signal in untreated cells by approximately 10- to 180-fold compared to the whole-cell assay (Figures 2A–2C). Conversely, *E. coli*, which is efficiently lysed by the BacTiter-Glo lysis buffer, showed a decrease in ATP signal with vancomycin treatment in both assays, and the ATP signal was not increased by bead beating (Figure 2D).

We then assessed the generalizability of our results by testing DprE1 and MmpL3 inhibitors, as prior reports show that MmpL3 inhibitors induce an ATP burst in *Mtb*.^15,17^ MmpL3 inhibitors of different chemical scaffolds (SQ109, AU1235, BM212, and NITD-349), as well as DprE1 inhibitors in clinical development (AZ7371 and BTZ043), showed a ≥10-fold ATP increase in the whole-cell BacTiter-Glo assay in agreement with previous reports.^15,17^ This was accompanied by approximately 2- to 6-fold decreases in ATP in the beat assay, demonstrating that the ATP-burst artifact extends across different classes of cell-wall-targeting compounds (Figure S4).

Collectively, these findings demonstrate that the ATP burst observed in the BacTiter-Glo whole-cell assay is an experimental artifact. In the absence of prior weakening of the cell wall by drugs, the BacTiter-Glo lysis buffer alone is insufficient to effectively lyse mycobacterial cells. This has important implications for all applications of this assay in mycobacteria, as cell wall integrity and permeability can vary depending on growth conditions and phase,^34^ making assay results highly dependent on cell wall status. To overcome this limitation and minimize bias introduced by incomplete lysis, mechanical disruption by bead beating provides a more reliable method when using this assay.

### Whole-cell ATP screening identifies cell wall synthesis inhibitors

**Figure 3.**
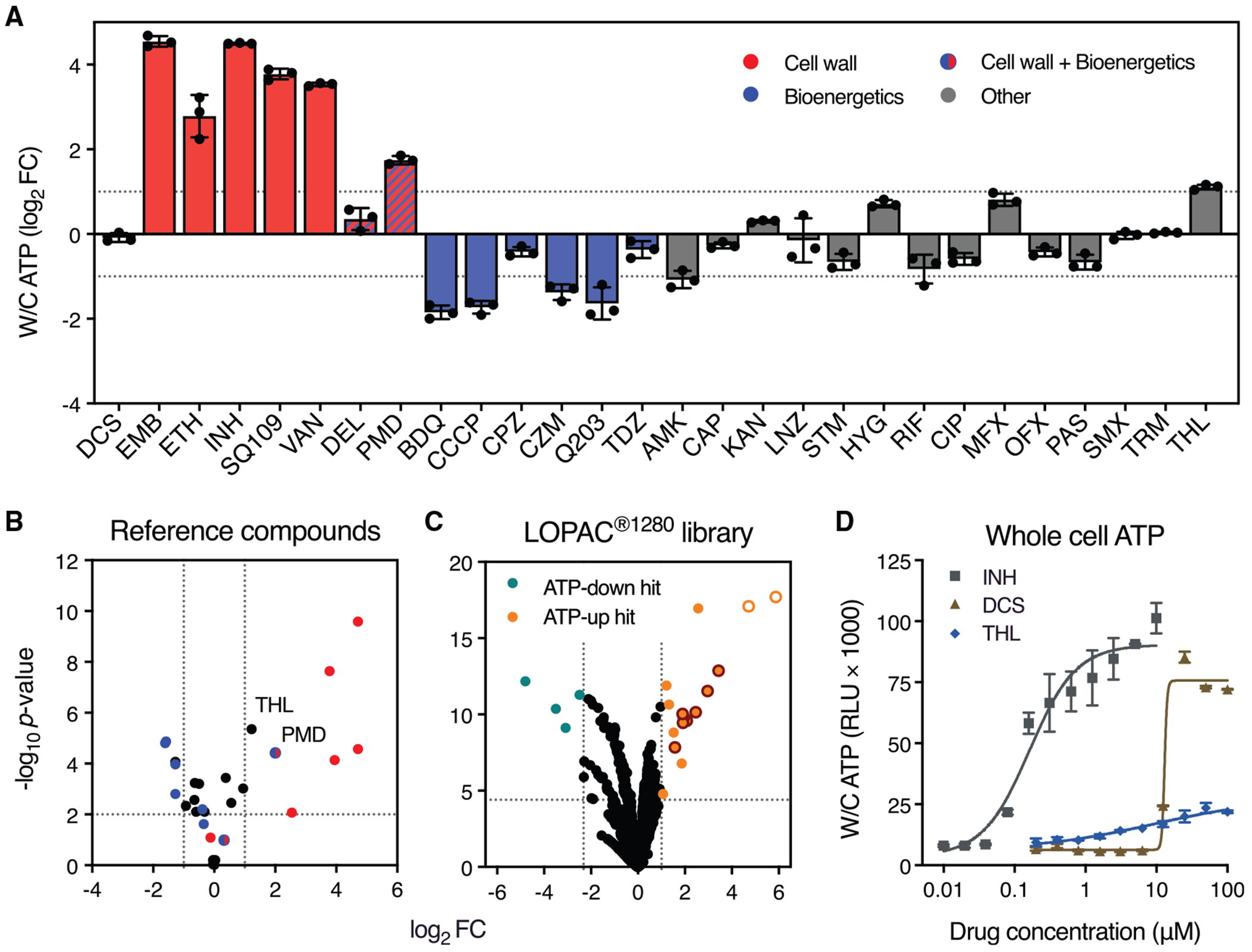
High-throughput ATP screening identifies compounds that target the *Mtb* cell wall (A and B) *Mtb* mc^2^6230 at OD 0.033 was treated for 24 h with 28 reference compounds (see Table 1) at 10 μM alongside DMSO-only controls, and ATP was measured using the BacTiter-Glo whole-cell assay. (A) Log_2_ fold changes relative to DMSO-only. Mean ± SD, *n* = 3 replicate wells. (B) Volcano plot for the experiment in (A). The dotted lines indicate a 2-fold change and a *p* value of 0.01. See also Figure S5. (C) Volcano plot showing responses relative to DMSO-only controls for the Sigma LOPAC1280 compound library screened using the same experimental setup in (A–B). The dotted lines indicate a 2-fold change for ‘ATP-up’ hits, an 80% decrease for ‘ATP-down’ hits, and a *p* value of 4 × 10^−5^ (Bonferroni cutoff, α = 0.05). Orange circles with dark red borders denote known cell wall inhibitors, and hollow orange circles denote compounds structurally related to ATP. See also Table 2. (D) Dose-response curves for isoniazid (INH), D-cycloserine (DCS), and tetrahydrolipstatin (THL). *Mtb* mc^2^6230 at OD 0.033 was treated for 24 h with increasing concentrations of each drug, and ATP was measured using the BacTiter-Glo whole-cell assay. Mean ± SD, *n* = 3 replicate wells. This experiment was repeated with similar results.

Having established the mechanistic basis of the ATP burst, we hypothesized that this response could serve as a functional assay to identify compounds that target the mycobacterial cell wall. In a proof-of-principle experiment, we screened a panel of 28 compounds with diverse mechanisms of action to confirm the specificity of the ATP burst to cell-wall-targeting drugs and to adapt the assay for high-throughput. These included inhibitors of DNA replication, transcription, translation, folate biosynthesis, cell wall biosynthesis, and bioenergetics (Table 1). *Mtb* mc^2^6230 was treated with drugs at 10 μM, and ATP was measured using the BacTiter-Glo whole-cell assay. After 24 h, a strong ATP signal increase (>4-fold) specific to cell-wall-targeting drugs was observed (Figures 3A, 3B, and S5), consistent with previous reports.^4^ Bioenergetics-targeting drugs showed their strongest effect at 48 h (Figure S5). The only cell wall drug not producing an ATP burst at 10 μM was D-cycloserine; however, at higher concentrations, an ATP burst was observed (Figure 3D). We also observed a significant ATP increase with the serine esterase inhibitor tetrahydrolipstatin (THL) (Figures 3A and 3B). THL has been reported to target lipid esterases involved in cell wall biosynthesis such as Pks13, TesA, and Ag85C^35,36^ and has been shown to reduce levels of the cell wall lipids PDIM, SL-1, and TDM.^23,37^ However, unlike isoniazid and D-cycloserine, THL did not produce a strong sigmoidal dose-response curve (Figure 3D), consistent with its broad nonspecific activity.^35,36^

**Figure 4.**
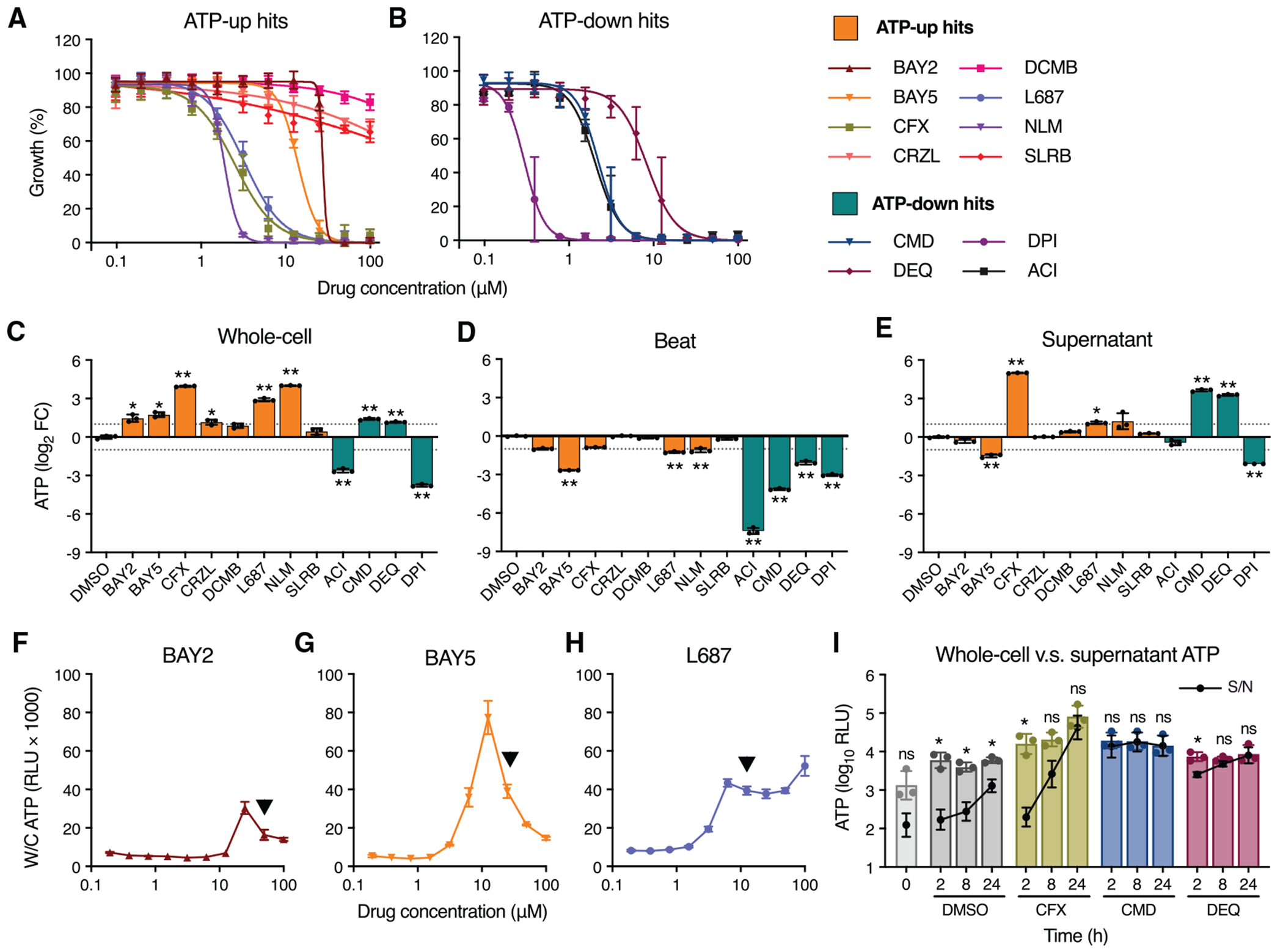
Validation of ATP screening hits from the LOPAC compound library (A and B) MIC assays for *Mtb* mc^2^6230 with selected LOPAC library hits (see Table 2). Growth was measured by OD_600_ and normalized to no-drug control wells. Mean ± SD, *n* = 4 replicate wells from two independent experiments. See also Figure S7. (C–E) Follow-up of ATP responses to LOPAC hits. Cultures at OD_600_ 0.033 were treated for 24 h at 20 μM, and ATP was measured using the BacTiter-Glo whole-cell assay (C), the bead-beating assay (D), and in the culture supernatant (E). Mean ± SD, *n* = 3 replicate cultures. *p < 0.05, **p < 0.001, and >2-fold change by unpaired *t* test with Holm-šídák’s MCT. (F–H) ATP whole-cell assay dose-response curves for Bay 11–7082 (F), Bay 11-7085 (G), and L-687,384 (H). Black arrows indicate the MIC_90_. Mean ± SD, *n* = 3 replicate wells. (I) Time course of ATP responses in the BacTiter-Glo whole-cell assay (bars) and in the culture supernatant (‘S/N,’ black circles and line) following treatment with 20 μM cefotaxime (CFX), calmidazolium (CMD), or dequalinium (DEQ). Mean ± SD, *n* = 3 means from replicate experiments performed in either duplicate or triplicate. *p < 0.05 and >2-fold change between supernatant and whole-cell ATP signal by unpaired *t* test with Holm-šídák’s MCT. For experiments in (C–I), cultures at OD_600_ 0.033 were treated for 24 h or for the duration indicated in (I). All experiments were repeated with similar results.

We then tested the feasibility of this approach using the Sigma LOPAC1280 library of 1,280 pharmacologically active compounds. This screen identified 16 compounds that induced a >2-fold increase in the ATP signal (“ATP-up” hits) (Figure 3C; Table 2). Seven of these hits were cephalosporins, which are β-lactam antimicrobials that inhibit peptidoglycan biosynthesis, validating our approach. Furthermore, the hit compound nialamide is a derivative of isoniazid and accordingly showed cross-resistance in an isoniazid-resistant mutant (Figure S6). Two compounds, P1,P4-di(adenosine-5^′^)tetraphosphate ammonium and 2-chloroadenosine triphosphate tetrasodium, were structurally related to ATP and increased the BacTiter-Glo signal independent of *Mtb* and were therefore excluded from further analyses. This left six ATP-up hits not previously known to target the cell wall. We assessed the ability of these compounds to inhibit *Mtb* growth, along with nialamide, the cephalosporin cefotaxime, and four compounds that were found to lower ATP by >80% (“ATP-down” hits) (Figure 3C; Table 2). Three of the six ATP-up hits—Bay 11-7082, Bay 11-7085, and L-687,384—as well as nialamide, cefotaxime, and all four ATP-down hits, inhibited the growth of both *Mtb* mc^2^6230 (Figures 4A and 4B) and virulent *Mtb* H37Rv (Figure S7), with MIC_90_ concentrations between 0.8 and 50 μM, demonstrating the ability of this approach to identify compounds with antitubercular activity in virulent and avirulent strains of *Mtb*. Furthermore, six of these compounds—nialamide, L-687,384, and all four ATP-down hits—have previously been shown to inhibit *M. bovis* BCG^38^ (Table 2). Antibacterial compounds in the library with other known mechanisms, such as doxycycline and ofloxacin, which target protein synthesis and DNA gyrase, respectively, did not come up as hits in our screen, highlighting the specificity of the setup to identify cell-wall-targeting compounds.

We next followed-up on these compounds by measuring ATP levels using our bead-beating assay to confirm that they indeed weaken the cell wall rather than increase ATP through an unrelated mechanism. The three ATP-up compounds that inhibited *Mtb* growth showed a >2-fold ATP increase in the whole-cell assay, which was accompanied by an ATP decrease in the beat assay, consistent with impaired cell wall integrity and increased ATP release (Figures 4C and 4D). The three compounds that failed to inhibit growth showed a weak or no response in the whole-cell assay follow-up and mirrored DMSO in the beat assay, consistent with the absence of antimycobacterial activity. Interestingly, unlike classical cell wall inhibitors, which produced strong sigmoidal dose-response curves (Figure 3D), Bay 11-7082 and Bay 11-7085 exhibited dose-response peaks at 0.5 × MIC_90_, with Bay 11-7082 showing a notably smaller maximal response (Figures 4F and 4G). This behavior suggests that these compounds might have multiple modes of action or, alternatively, may reflect reduced viability at higher drug concentrations. We note that these two compounds, together with those lacking antimycobacterial activity and several cephalosporins, showed weaker responses in the initial screen (2- to 4-fold changes) (Table 2). This suggests that a 2-fold detection threshold may sit near the cutoff between meaningful activity and noise, while still detecting compounds with weak cell-wall-targeting effects or low potency.

Surprisingly, in our follow-up assay, two of the ATP-lowering hits—calmidazolium (CMD) and dequalinium (DEQ)—increased the ATP signal in the whole-cell assay, though it was substantially lowered in the beat assay (Figures 4C and 4D). Both compounds also rapidly increased supernatant ATP levels, indicating cell lysis (Figures 4E–4I). In agreement with our results, dequalinium is reported to induce a range of effects on microbial cells, including inhibition of the F_1_-ATPase,^39^ and has been suggested to affect cell membrane permeability, potentially leading to lysis.^40^ Cefotaxime also substantially increased supernatant ATP after 24 h, though not at earlier time points (Figures 4E–4I). These results demonstrate the application of the BacTiter-Glo assay for high-throughput identification of cell wall inhibitors as well as the versatility of this assay to probe the effects of antitubercular compounds on the *Mtb* cell wall and bioenergetics.

### Detection of antagonistic and synergistic interactions with cell wall drugs

Compounds that disrupt cellular bioenergetics result in reduced ATP production.^41^ Inhibitors of energy metabolism have been shown to antagonize cell-wall-targeting compounds, suppressing the ATP burst and reducing killing,^3,4,14,16^ leading some authors to conclude that excessive ATP contributes to the lethality of cell wall drugs.^4^ Consistent with these studies, we observed suppression of the isoniazid-induced ATP increase in the whole-cell assay and reduced killing following co-treatment with the bioenergetics inhibitors bedaquiline, Q203, and CCCP (Figures S8A and S8B). Conversely, the BacTiter-Glo beat assay showed decreased ATP in cultures treated with isoniazid alone, and this was further depressed by cotreatment with bioenergetics inhibitors (Figure S8C). Considering that the ATP burst reflects disruption of the mycobacterial cell wall (Figures 1 and 2), these data indicate that cotreatment with bioenergetics inhibitors leads to a reduction in the canonical activity of cell wall inhibitors rather than mitigating “death by ATP.”

**Figure 5.**
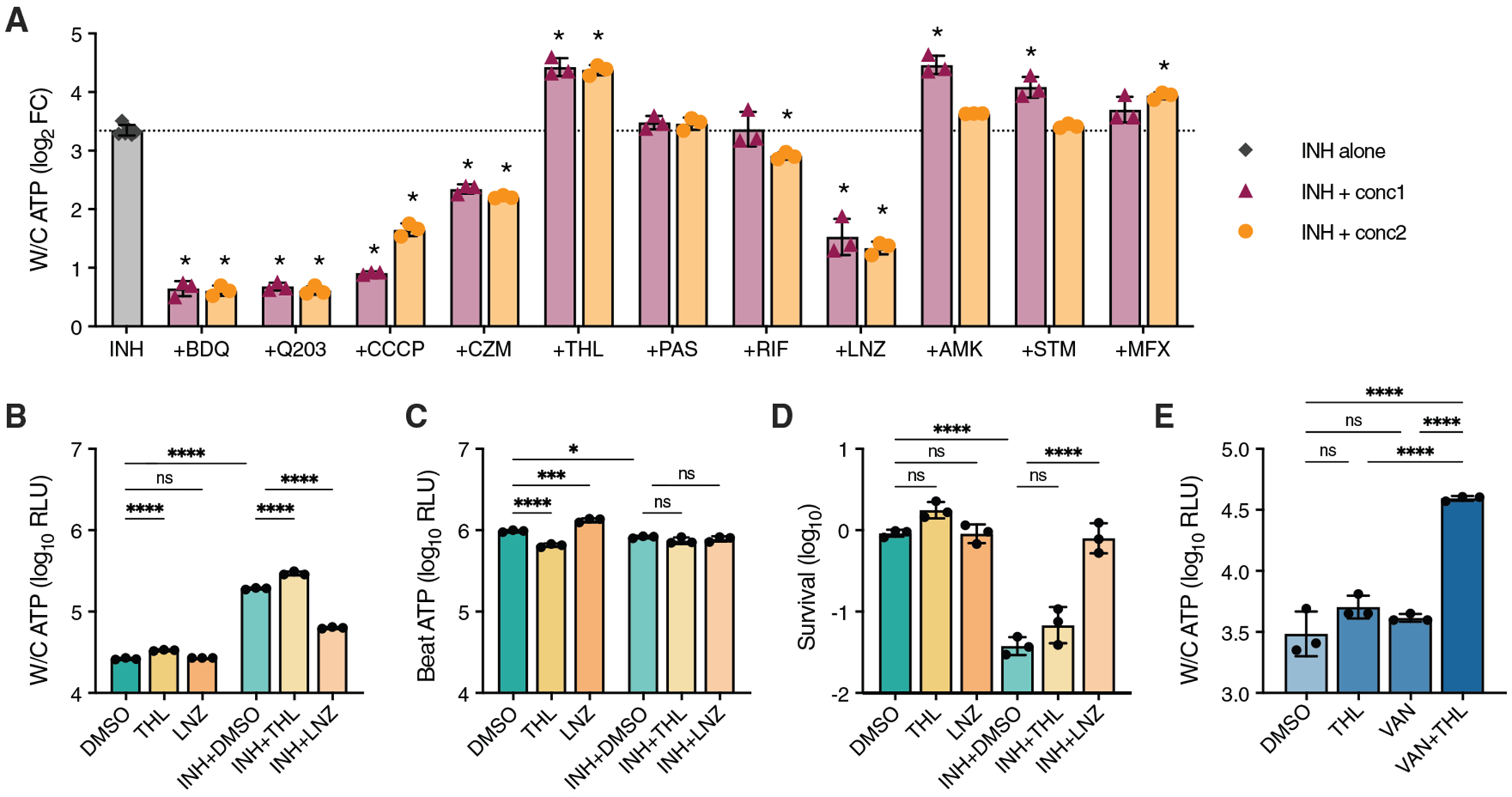
ATP screening identifies antagonistic and synergistic interactions with cell wall inhibitors (A) *Mtb* mc^2^6230 at OD 0.33 was treated for 24 h with isoniazid alone (INH, 0.1 μg/mL) or in combination with the indicated compounds at 2–4 × and 5–10 × MIC_90_ (‘conc1’ and ‘conc2,’ respectively) (see Tables 1 and S1). Cultures were incubated in 96-deep-well blocks with fast shaking to maximize growth (see Figure S9). ATP was measured using the BacTiter-Glo whole-cell assay. Data are shown as log_2_ fold changes compared to the DMSO-only control. Mean ± SD, *n* = 6 replicate wells for DMSO and isoniazid, and *n* = 3 for cotreatments. *p < 0.001 vs. isoniazid alone by two-way ANOVA with Dunnett’s MCT. This experiment was performed once. (B–D) Follow-up of putative antagonistic and synergistic effects identified in (A). Cultures at OD 0.33 were treated for 24 h with isoniazid (INH, 0.1 μg/mL), linezolid (LNZ, 2 μg/mL), and tetrahydrolipstatin (THL, 50 μg/mL) as indicated, and ATP was measured using the BacTiter-Glo whole-cell (B) and beat assays (C) and plated for CFU to assess viability (D). The starting cell density was 3 × 10^8^ CFU/mL. Mean ± SD, *n* = 3 replicate cultures. *p < 0.05, ***p < 0.001, and ****p < 0.0001 by one-way ANOVA with šídák’s MCT. (E) Cotreatment with tetrahydrolipstatin and vancomycin. PDIM(+) *Mtb* mc^2^6230 at OD 0.033 was treated with 1 μg/mL of each drug alone and in combination for 24 h, and ATP was measured using the BacTiter-Glo whole-cell assay. Cultures were treated in 96-well microtiter plates and supplemented with 0.1 mM propionate to support PDIM production. See also Figure S11. ****p < 0.0001 by one-way ANOVA with šídák’s MCT. Mean ± SD, *n* = 3 replicate wells. The experiments in (B–E) were repeated with similar results.

We speculated that this antagonistic interaction could be partly related to general metabolic slowdown, in which case it may not be specific to bioenergetics inhibitors. To test this, we cotreated *Mtb* mc^2^6230 with isoniazid and 11 compounds with varying modes of action (Tables 1 and S1). To improve experimental throughput, we used 96-deep-well blocks incubated with vigorous shaking, which supports much faster *Mtb* replication rates than conventional 96-well microtiter plates (Figure S9). Cotreatment with bioenergetics inhibitors (bedaquiline, Q203, CCCP, and clofazimine), as well as the protein synthesis inhibitor linezolid, substantially reduced the ATP signal at both doses tested compared to isoniazid alone, while THL significantly increased it (Figure 5A), pointing to potential antagonistic and synergistic effects, respectively. We followed-up on these interactions by comparing ATP levels in the BacTiter-Glo whole-cell and beat assays and plated for viability. Linezolid cotreatment significantly decreased isoniazid-induced ATP release and abrogated killing, resulting in no significant difference in survival compared to the DMSO control (*p* = 0.9997) and a 1.3-log reduction in killing compared to isoniazid alone (*p* < 0.0001), consistent with an antagonistic effect (Figures 5B–5D). This interaction was not detected in the checkerboard assay (FICI = 1.0, Table S2); thus, this effect would be missed if only the checkerboard assay was performed. Unlike bioenergetics inhibitors, which lowered ATP in the beat assay (Figure S8C), linezolid alone resulted in a small but significant increase in ATP (1.4-fold, *p* = 0.0002), and cotreatment did not further lower ATP compared to isoniazid alone (Figure 5C). Given that protein biosynthesis is one of the most energetically costly cellular processes,^42^ this increase may result from an immediate reduction in ATP consumption upon translation arrest. In contrast, THL cotreatment significantly increased ATP in the whole-cell assay compared to isoniazid alone, with no difference in the beat assay, indicating greater ATP release in the cotreatment; however, this did not translate to an effect on killing (Figures 5B–5D). We again saw a small but significant increase in ATP in the whole-cell assay for THL alone (Figures 3 and 5B), suggesting this may be an additive effect, consistent with what we observed in the checkerboard assay (FICI = 0.71, Table S2). Furthermore, isoniazid alone kills *Mtb* very rapidly^43^; therefore, further improvement of isoniazid-induced killing might not be possible at the dose used. Nevertheless, combination treatment could have the potential to prevent the emergence of isoniazid resisters.

Next, we determined if the status of the cell wall lipid phthio-cerol dimycocerosate (PDIM) impacts our findings. Since beginning this work, we have established that our *Mtb* mc^2^6230 stock is a predominantly PDIM-negative mixed population and have re-isolated a pure PDIM-positive clone.^26^ In PDIM-positive *Mtb* mc^2^6230, isoniazid induced an artifactual ATP burst in the BacTiter-Glo whole-cell assay, both in standard 7H9 and propionate-supplemented PDIM maintenance media^26^ (Figure S10). These results verify the applicability of this approach to PDIM-positive strains.

Finally, as a proof of concept, we used this approach to detect a previously described synergy between THL and vancomycin.^23^ Vancomycin is a large antibiotic whose efficacy is significantly affected by cell wall permeability and PDIM levels.^22,25,26^ THL synergizes with vancomycin by lowering PDIM production and impairing cell wall integrity.^23^ As both compounds cause an ATP burst, we first determined concentrations of vancomycin and THL that produced minimal effects on the BacTiter-Glo whole-cell ATP signal in PDIM-positive *Mtb* (Figure S11). We found that at 1 μg/mL, both drugs induced only a ~1.5-fold increase compared to the DMSO control; however, when used in combination, the ATP signal increased 13-fold, denoting a strong potentiation of cell wall effects (Figure 5E). This is consistent with previous work showing synergism between THL and vancomycin^23^ and demonstrates the potential of this approach to screen for such interactions.

## DISCUSSION

One key distinguishing feature of mycobacteria is their complex cell wall. This contributes to their intrinsic resistance to many antibiotics and their ability to persist in the host and makes them notoriously difficult to lyse.^34^ As such, commercial kits that work well on other species with less complex cell walls, such as Gram-negative bacteria, are much less efficient against mycobacteria, which are better lysed using more aggressive mechanical methods, such as bead beating or heat inactivation.^27–29^ Accordingly, we discovered that the previously reported ATP burst phenomenon^3,4,11–16^ is an artifact of improved cell lysis facilitated by drugs that weaken the cell wall. This is in line with Koul et al.,^5^ who did not observe an increase in ATP in *Mtb* after 24 h of isoniazid treatment using heat inactivation and an ATP bioluminescence assay kit. While our findings highlight the need for mechanical lysis to improve the accuracy and reliability of BacTiter-Glo-based ATP measurements in mycobacteria, they also reveal a valuable research application of the ATP burst phenomenon. A successful example employing this approach to identify potent cell wall inhibitors against *M. abscessus* is described in the companion paper by Singh et al.^44^ Our data establish a direct link between the ATP burst and the canonical mechanism of action of cell wall inhibitors, providing a rapid functional readout that can be leveraged in several ways: (1) to assess whether compounds with unknown mechanisms affect the mycobacterial cell wall; (2) as a high-throughput screening tool to identify new cell wall inhibitors from large compound libraries; and (3) to evaluate potential synergistic or antagonistic interactions with known cell-wall-targeting drugs. An increase in ATP relative to controls (typically >2- to 4-fold) can be interpreted as evidence of a weakened cell wall and subsequently validated using the complementary bead-beating assay to quantify ATP in fully lysed cultures. Additional analyses, such as measuring extracellular ATP and generating dose-response curves, can further support and refine conclusions regarding cell-wall-targeting activity. In optimizing our screen, we found that while cell-wall-targeting effects were best captured after 24 h, the strongest response for bioenergetics inhibitors was observed after 48 h. To enhance the assay’s utility as a dual-purpose screen, an additional later time point could be included to better capture ATP-lowering effects. However, extended incubation may increase the potential for confounding factors, such as variability due to differences in replication rates and cell death.

Our bead-beating protocol presents an approach to use the BacTiter-Glo kit to more reliably assess ATP levels in mycobacterial cultures by removing the bias of incomplete lysis. This approach may also increase assay sensitivity when assessing bioenergetic inhibitors, as incomplete lysis reduces the effective dynamic range. However, for a more detailed assessment of cellular bioenergetics, it may be necessary to determine the ratio of ATP, ADP, and AMP, as this provides a better representation of functional energy homeostasis than ATP level alone.^45^ This can be done, for example, by mass spectrometry.^46^ Alternatively, in recent years, there have been several reports using genetically encoded biosensors to measure mycobacterial ATP abundance^47–49^ or the ATP/ADP ratio.^50,51^ This enables realtime ATP(/ADP) tracking at the single-cell level with improved spatiotemporal resolution and avoids normalization challenges related to differences in biomass and cell viability. While these assays reliably detect reduced ATP levels in response to bioenergetic inhibitors,^47,48,50^ their performance with isoniazid has been inconsistent, with studies reporting ATP depletion in *M. smegmatis*,^48^ increased ATP in *Mtb*,^47^ and no effect on the ATP/ADP ratio in *Mtb*.^50^ This might be due to the use of different sensors in these studies, different strains, varying assay conditions, or sensitivity issues. Further improvement and standardization of ATP biosensors will provide a valuable tool for studying bacterial bioenergetics.

Our findings indicate that bioenergetics inhibitors reduce the canonical cell-wall-targeting activity of isoniazid. This is consistent with a model whereby drug-induced ATP depletion renders cells more tolerant to isoniazid by decreasing target activity, rather than by inhibiting a toxic ATP surge. Given the substantial energetic cost of cell wall biosynthesis, we speculate that impaired ATP production could limit these processes, thereby blunting the effectiveness of cell wall drugs. Furthermore, we have previously shown that bacteriostasis alone is insufficient to protect against isoniazid-induced killing.^14^ Collectively, these observations highlight that antagonism of isoniazid is complex and multifactorial, and the mechanistic basis underlying these effects warrants further investigation.

Independent of the specific mechanisms involved, our work demonstrates how whole-cell ATP luminescence screening can be employed to detect synergistic and antagonistic drug interactions with cell wall inhibitors. We anticipate that this could be further expanded, for example, to screen for nutrient or genetic interactions. This approach also enabled us to detect interactions missed by the conventional checkerboard assay. A similar discordance between bactericidal activity and growth inhibition has been reported in *M. abscessus* for bedaquiline and β-lactams, which also have an antagonistic interaction in the BacTiter-Glo whole-cell assay.^16^ The ability to screen for synergistic interactions could be particularly useful for identifying combinations with a clear mechanistic rationale, such as improving the potency of cell wall drugs whose activity is limited, for example, due to degradation (e.g., β-lactams) or poor uptake (e.g., vancomycin). β-lactams are a largely untapped resource for mycobacterial treatment, primarily due to the presence of chromosomally encoded β-lactamases that confer intrinsic resistance.^52^ Furthermore, in addition to canonical D,D-transpeptidases, most peptidoglycan cross-links in mycobacteria are 3→3 linkages catalyzed by non-classical L,D-transpeptidases, which are generally not inhibited by these drugs.^53^ Approaches to circumvent these limitations include using β-lactamase inhibitors^54,55^ or combinations of different β-lactam subclasses to target both L,D- and D,D-transpeptidases.^56^ ATP screening could provide a simple, high-throughput method for identifying β-lactam combinations, potentially aiding the development of more effective tuberculosis and NTM treatment regimens.

Finally, the high-throughput cultivation method developed in this work, enabling *Mtb* to be grown in 96-well plates at growth rates comparable to inkwells or roller bottles, will be a valuable tool for tuberculosis research in general.

In conclusion, our work provides a mechanistic explanation for the ATP burst detected with the BacTiter-Glo kit in the presence of cell-wall-targeting compounds, directly linking this phenomenon to the canonical mode of action of these drugs. We show that the simplicity, speed, low cost, and high-throughput compatibility of this approach make it a valuable addition to the antimycobacterial drug discovery and development toolkit. Moreover, we propose practical solutions to more accurately measure ATP levels in mycobacteria by addressing the confounding effects of incomplete cell lysis.

### Limitations of the study

While we demonstrate that the widely reported ATP burst with the BacTiter-Glo assay is a lysis artifact, the true intracellular ATP response to cell wall drugs remains to be established. Current data from biosensor studies present contradictory outcomes. While a relative ATP increase in response to isoniazid has been reported in one biosensor study,^47^ other studies have observed the opposite outcome or no effect.^48,50^ Further development and implementation of ATP biosensors will be valuable for fully clarifying intracellular ATP responses. Second, while our approach provides a simple means to rapidly screen for potential antagonistic or synergistic interactions *in vitro*, such interactions do not always predict *in vivo* responses.^57^

## RESOURCE AVAILABILITY

### Lead contact

Requests for further information and resources should be directed to and will be fulfilled by the lead contact, Michael Berney (michael.berney@einsteinmed.edu).

### Materials availability

Pure PDIM(+) *Mtb* strains used in this study are available from the lead contact upon completion of a materials transfer agreement.

## STAR★METHODS

### EXPERIMENTAL MODEL AND STUDY PARTICIPANT DETAILS

The following bacterial strains were used in this study: *Mycobacterium tuberculosis* mc^2^6230,^30^ *M. tuberculosis* mc^2^6230 AE1601 (PDIM-positive clone),^26^ *M. tuberculosis* H37Rv AE1001 (PDIM-positive clone; this study), *Mycobacterium abscessus* (ATCC 19977), *Mycobacterium smegmatis* mc^2^155,^58^ and *Escherichia coli* K-12 (ATCC 10798). Bacterial strains were cultured and maintained under standard laboratory conditions appropriate for each species in accordance with institutional biosafety guidelines. No human participants or vertebrate animals were used in this study; therefore, sex, gender, age, ancestry, and ethnicity are not applicable, and no institutional ethical approvals (IRB or IACUC) were required. As only microbial strains were used, sex as a biological variable is not applicable. No cell lines were used in this study.

### METHOD DETAILS

#### Bacterial strains and culture conditions

*Mtb* mc^2^6230 (H37Rv Δ*RD1* Δ*panCD*)^30^ and *M. smegmatis* mc^2^155^58^ strains were obtained from laboratory stocks. A pure PDIM-positive mc^2^6230 clone (AE1601) was previously isolated from our mc^2^6230 stock.^26^ PDIM-positive *Mtb* H37Rv (AE1001) was isolated following C57BL/6 mouse passage using previously described methods.^26^ *M. abscessus* (ATCC 19977) and *E. coli* K-12 (ATCC 10798) were obtained directly from ATCC. *Mtb* strains were cultured in Middlebrook 7H9 broth supplemented with 10% (v/v) OADC (0.6 g/L sodium oleate, 50 g/L bovine serum albumin fraction V, 20 g/L dextrose, 40 mg/L catalase, 8.5 g/L sodium chloride), 0.2% (v/v) glycerol, and 0.05% (v/v) tyloxapol, with the addition of 24 mg/L D-calcium pantothenate for *Mtb* mc^2^6230. PDIM-positive strains were additionally supplemented with 0.1 mM sodium propionate to maintain and enhance PDIM production.^26^ *M. abscessus* and *M. smegmatis* were cultured in Middlebrook 7H9 broth supplemented with 10% (v/v) Middlebrook ADC enrichment, 0.2% (v/v) glycerol, and 0.05% (v/v) Tween 80. Middlebrook 7H10 agar supplemented with 10% (v/v) OADC and 0.5% (v/v) glycerol was used as a solid medium for plating mycobacterial strains, with the addition of 24 mg/L D-calcium pantothenate for *Mtb* mc^2^6230. *E. coli* K-12 was cultured in LB broth, and LB agar was used for plating. For *Mtb* experiments, fresh starter cultures were inoculated from frozen seed stocks, and for *M. abscessus, M. smegmatis*, and *E. coli*, single colonies were picked from agar plates. All strains were sub-cultured once, grown to logarithmic phase (OD_600_ ~0.8), and then diluted to the required density for each experiment. Mycobacterial cultures were grown at 37°C with gentle shaking at 100 rpm for BSL2 strains and 80 rpm for BSL3. *E. coli* was incubated at 37°C with shaking at 200 rpm.

#### BacTiter-Glo ATP assays

The BacTiter-Glo Microbial Cell Viability Assay (Promega) was used to quantify ATP following treatment with antimicrobial compounds. The total DMSO concentration was kept ≤1% for all experiments. For whole-cell ATP assays, 25 μL of bacterial culture and 25 μL of the BacTiter-Glo reagent were combined in 96-well white opaque plates. Plates were incubated in the plate reader in the dark with shaking for 5 min, and then luminescence was measured using either a FLUOstar Omega or VANTAstar Microplate Reader (BMG Labtech). The LOPAC screen was measured using a Cytation-3 Cell Imaging Multiple-Mode Reader (LabX) with a 1 s integra-tion to capture luminescence. To pre-lyse cells by bead beating (‘beat’ assay), 250 μL of bacterial culture was added to approximately 250 μL of 0.1 mm zirconia/silica beads (BioSpec) and then lysed using a Precellys Cryolys Evolution bead beater (Bertin Technolo-gies) cooled to 0°C for three 20 s cycles at 6,800 rpm with a 30 s pause between cycles. Lysed samples were boiled at 100°C for 5 min to denature cellular enzymes, then briefly cooled on ice before centrifugation at 4°C for 5 min at 13,000 rpm. Heat lysis (‘heat’ assay) was performed by boiling 250 μL culture for 30 min at 100°C and then pelleting as above. 25 μL of the lysate from either the beat or heat extractions and 25 μL of the BacTiter-Glo reagent were added to white 96-well plates, which were incubated and measured as for whole-cell samples. To quantify supernatant ATP, 250 μL of bacterial culture was centrifuged for 3 min at 13,000 rpm. The supernatant was then passed through a 0.22 μm filter, and ATP in the filtrate was measured as for whole-cell culture and lysates. Calibration curves of ATP chemical standards (Sigma) prepared in water were used to determine the linear range.

#### ATP quantification by LC-MS

*Mtb* mc^2^6230 cultures at OD_600_ 0.33 were treated with antimicrobial compounds as specified for 24 h in 10 mL inkwell bottles, after which 8 mL of culture was pelleted by centrifugation at room temperature for 5 min at 2,600 × *g*. The cell pellet was resuspended in 1 mL ice-cold extraction solvent containing 20:40:40 (v/v) LC-MS grade water/acetonitrile/methanol. This was then transferred to approximately 500 μL of 0.1 mm zirconia/silica beads (BioSpec) and lysed by bead beating as above. Samples were centrifuged, and the extracts filtered through a 0.22 μm Nylon Spin-X microcentrifuge filter (Corning) and stored at −80°C until analysis. ATP quantification was performed using an Agilent 1290 Infinity II liquid chromatography system coupled with an Agilent 6545 quadrupole time-of-flight (Q-TOF) mass spectrometer equipped with a Dual Agilent Jet Stream Electrospray Ionization (Dual AJS ESI) source operated in negative mode. Metabolites were separated on an InfinityLab Poroshell 120 HILIC-Z, 2.1 × 150 mm, 2.7 μm, 100 Å col-umn (Agilent) as previously described.^26,59^ Mass spectra were recorded in profile mode from *m*/*z* 60 to 1200 using an acquisition rate of 1 spectra/sec in the 2 GHz extended dynamic range mode and 1700 *m*/*z* low mass range, using the sensitive slicer mode. The ESI and Q-TOF settings were optimized for the detection of ATP and were set as follows: gas temperature 350°C, gas flow 13 L/min, sheath gas temperature 350°C, sheath gas flow 12 L/min, and the capillary, nozzle, fragmentor, skimmer, and octopole voltages were 3500, 2000, 150, 45, and 750 V, respectively. Dynamic mass axis calibration was achieved by continuous infusion of a reference mass solution using an isocratic pump with a 100:1 splitter. HPLC-grade water (Cen-Med Enterprises) and LC-MS grade solvents (Fisher Chemical) were used for both the LC-MS mobile phase and metabolite extraction. Data analysis was performed using the Agilent MassHunter Qualitative (v10.0) and Quantitative Analysis Software (v10.1). ATP identification was based on mass retention times determined using a chemical standard and isotope distribution patterns, and quantified by integrating the area under the curve using a mass tolerance of 20 ppm. Calibration curves of ATP standards in extraction buffer and spiked into a homologous mycobacterial extract were used to determine the linear range and check for ion suppression effects.

### Drug resistance and viability assays

Resistance of *Mtb* strains to LOPAC hits and other compounds was assessed using the microbroth dilution method to determine MICs. Assays were prepared in flat-bottom 96-well microtire plates by either performing 2-fold serial dilutions in media at 2 × final drug concentration, or by spotting 2 μL of a 100 × drug dilution in DMSO followed by the addition of 98 μL of media. Log-arithmic-phase subcultures were diluted to OD_600_ 0.01, and 100 μL was then added to the plate to give a final OD_600_ of 0.005 and 1 × drug concentration. Only the inner wells were used for assay, and the outer wells were aliquoted with 200 μL PBS or media. Checkerboard assays were prepared by spotting 2 μL of a 100 × dilution series of either linezolid or THL in DMSO down the plate columns and a 100 × dilution series of isoniazid in water across the rows, creating a two-dimensional matrix. Media and cells were then added as for MIC assays. Plates were incubated at 37°C with gentle shaking (80–100 rpm), and bacterial growth was measured after 10 days by OD_600_ using a microplate reader. MIC data were normalized to drug-free control wells and fit with non-linear regression in GraphPad Prism 10. The MIC was defined as the concentration in the well that resulted in 90% inhibition of bacterial growth. The fractional inhibitory concentration index (FICI) for checkboard assays was calculated as follows: FICI = (MIC_AB_/MIC_A_) + (MIC_BA_/MIC_B_).^60^ FICI values were calculated using wells closest to half the MIC for each drug, and the mean value reported.

Bacterial viability before and after drug exposure was determined by plating 100 μL of 1:10 serial dilutions in PBS with 0.05% ty-loxapol onto agar. Colonies were counted after 3–4 weeks of incubation at 37°C.

### QUANTIFICATION AND STATISTICAL ANALYSIS

Statistical analyses were performed using GraphPad Prism 10. Significance was determined by unpaired *t* tests or ordinary one- or two-way ANOVA with testing for multiple comparisons as specified in the figure legends. CFU and ATP data were log_10_ transformed as indicated to improve normality and homogeneity of variance. The Brown-Forsythe and Kolmogorov-Smirnov tests, as well as the homoscedasticity and QQ plots, were used to assess the effects of data transformation and test assumptions.

## Supplementary Material

1

Supplemental information can be found online at https://doi.org/10.1016/j.celrep.2026.117286.

## Figures and Tables

**Table 1.** Reference compounds included in this study

MoA category	Compound	Abbr.
Cell wall	D-cycloserine	DCS
	ethambutol	EMB
	ethionamide	ETH
	isoniazid	INH
	SQ109	–
	vancomycin	VAN

Bioenergetics	bedaquiline*	BDQ
	CCCP*	–
	chlorpromazine	CPZ
	clofazimine*	CZM
	Q203*	–
	thioridazine	TDZ

Bioenergetics + cell wall	delamanid	DEL
	pretomanid	PMD

Translation	amikacin*	AMK
	capreomycin	CAP
	hygromycin B	HYG
	kanamycin	KAN
	linezolid*	LNZ
	streptomycin*	STM

Transcription	rifampicin*	RIF

DNA replication	ciprofloxacin	CIP
	moxifloxacin*	MFX
	ofloxacin	OFX

Folate biosynthesis	4-aminosalicylic acid*	PAS
	sulfamethoxazole	SMX
	trimethoprim	TRM

Lipid metabolism/other	tetrahydrolipstatin*	THL

All 28 compounds were included in the whole-cell ATP screen (Figures 3A and 3B).

The 11 compounds indicated with an asterisk (*) were included in the cotreatment screen with isoniazid (Figure 5A).

MoA, mode of action; Abbr., abbreviation.

**Table 2.** Sigma LOPAC1280 compound library whole-cell ATP screening hits

ATP	Abbr.	Compound	Target/comment	FC	*p*-value
Up	–	P1,P4-di(adenosine-5′)tetraphosphate ammonium	structurally related to ATP	59.0	2.0 × 10^18^
Up	–	2-chloroadenosine triphosphate tetrasodium	structurally related to ATP	26.5	8.2 × 10^18^
Up	NLM	nialamide^a^	monoamine oxidase inhibitor^b^; isonicotinic acid derivative	10.9	1.4 × 10^13^
Up	CFX	cefotaxime sodium salt	third-generation cephalosporin	7.84	2.8 × 10^12^
Up	L687	L-687,384 hydrochloride^a^	σ1 receptor agonist^b^	5.96	1.1 × 10^17^
Up	–	cephradine	first-generation cephalosporin	5.54	7.0 × 10^11^
Up	–	cefaclor	second-generation cephalosporin	4.25	2.6 × 10^10^
Up	–	ceftriaxone sodium salt	third-generation cephalosporin	3.90	1.5 × 10^10^
Up	–	cephalothin sodium salt	first-generation cephalosporin	3.80	3.4 × 10^10^
Up	–	cephalexin hydrate	first-generation cephalosporin	3.75	9.2 × 10^11^
Up	SLRB	salirasib	Ras inhibitor^b^; salicylic acid derivative	3.66	1.7 × 10^7^
Up	–	cefazolin sodium salt	first-generation cephalosporin	2.99	1.5 × 10^8^
Up	BAY5	Bay 11–7085	inhibitor of IκBα phosphorylation, preventing NF-κB activation^b^	2.87	1.5 × 10^9^
Up	CRZL	cirazoline hydrochloride	α1-adrenergic receptor agonist/α2-adrenergic receptor antagonist^b^	2.50	2.3 × 10^11^
Up	DCMB	(±)-2,3-dichloroalphamethylbenzylamine hydrochloride	PNMT (phenylethanolamine N-methyltransferase) inhibitor^b^	2.32	1.2 × 10^12^
Up	BAY2	Bay 11–7082	inhibitor of IκBα phosphorylation, preventing NF-κB activation^b^	2.11	1.6 × 10^5^
Down	DEQ	dequalinium chloride hydrate^a^	quaternary ammonium compound; broad-spectrum antibacterial and antifungal activity	0.18	5.0 × 10^12^
Down	CMD	calmidazolium chloride^a^	calmodulin antagonist^b^	0.12	7.4 × 10^10^
Down	ACI	AC-93253 iodide^a^	inhibitor of Src-family tyrosine kinases^b^	0.09	4.2 × 10^11^
Down	DPIC	diphenylene iodonium chloride^a^	flavoprotein inhibitor, including NADPH oxidase and nitric oxide synthase; broad-spectrum antibacterial and antifungal activity	0.04	6.5 × 10^13^

Small molecule hits identified in the BacTiter-Glo whole-cell assay that either significantly increased the ATP signal by > 2-fold (“ATP-up” hits) or significantly decreased the ATP signal by >80% (“ATP-down” hits). See also Figure 3C. Abbr., abbreviation; FC, fold change.

a Previously reported to inhibit the growth of *M. bovis* BCG (Kidwai et al.^38^).

b Mammalian-specific target/s

**Table:** KEY RESOURCES TABLE

REAGENT or RESOURCE	SOURCE	IDENTIFIER
Bacterial and virus strains		
*M. tuberculosis* mc^2^6230	Sambandamurthy et al.^30^	N/A
*M. tuberculosis* mc^2^6230 AE1601 (PDIM-positive clone)	Mulholland et al.^26^	N/A
*M. tuberculosis* H37Rv AE1001 (PDIM-positive clone)	This study	N/A
*M. abscessus*	ATCC	ATCC 19977
*M. smegmatis* mc^2^155	Snapper et al.^58^	N/A
*E. coli* K-12	ATCC	ATCC 10798
Chemicals, peptides, and recombinant proteins		
Bovine serum albumin fraction V	GoldBio	A-420-1
Catalase	Sigma-Aldrich	C1345
D-Calcium pantothenate	Acros Organics	243305000
Dextrose (D-glucose)	Fisher Chemical	D16
Glycerol	Fisher Chemical	G33
LB Agar	BD Difco	244520
LB Broth	BD Difco	244610
Middlebrook 7H10	BD Difco	262710
Middlebrook 7H9	BD Difco	271310
Middlebrook ADC Enrichment	BD Difco	211887
Sodium chloride	Fisher Chemical	S271
Sodium oleate	Strem Chemicals	11–1280
Sodium propionate	Sigma-Aldrich	P1880
Tween 80	Sigma-Aldrich	P1754
Tyloxapol	Sigma-Aldrich	T8761
Adenosine 5’-triphosphate disodium salt hydrate (ATP)	Sigma	A2383, A7699
Amikacin disulfate salt (AMK)	Acros Organics	455190050
AU1235	MedChemExpress	HY-101867
AZ7371	Cayman Chemical	19310
Bedaquiline (BDQ)	Gift from Kevin Pethe, Nanyang Technological University	N/A
BM212	MedChemExpress	HY-100725
BTZ043	MedChemExpress	HY-13579
Capreomycin Sulfate (CAP)	Alfa Aesar	J66684MD
Carbonyl cyanide 3-chlorophenylhydrazone (CCCP)	Tocris	0452
Ciprofloxacin (CIP)	Acros Organics	449620050
Chlorpromazine hydrochloride (CPZ)	MP Biomedicals, Inc.	190326
Clofazimine (CZM)	TCI	C2866
D-Cycloserine (DCS)	Acros Organics	228480050
Delamanid (DEL)	MedChemExpress	HY-10846
Ethambutol dihydrochloride (EMB)	Alfa Aesar	J60695
Ethionamide (ETH)	TCI	E0695
Hygromycin B (HYG)	Invitrogen	10687010
Isoniazid (INH)	Sigma	I-3377
Kanamycin Sulfate (KAN)	Fisher BioReagents	BP906
Linezolid (LNZ)	Acros Organics	460592500
Moxifloxacin hydrochloride (MFX)	Acros Organics	457960010
NITD-349	MedChemExpress	HY-109588
Ofloxacin (OFX)	Alfa Aesar	J62080
4-Aminosalicylic acid (PAS)	Acros Organics	104620050
Pretomanid (PMD)	MedChemExpress	HY-10844
Q203	Gift from Kevin Pethe, Nanyang Technological University	
Rifampicin (RIF)	Sigma	R3501
Sulfamethoxazole (SMX)	Fluka analytica-Sigma	S7507
SQ109	Sigma	SML1309
Streptomycin sulfate salt (STM)	Gold Biotechnology	S15050
Thioridazine hydrochloride (TDZ)	Tocris	3070
Tetrahydrolipstatin (Orlistat) (THL)	TCI	O0381
Trimethoprim (TRM)	Teknova	T1205
Vancomycin hydrochloride (VAN)	Alfa Aesar	J62790
Critical commercial assays		
BacTiter-Glo^™^ Microbial Cell Viability Assay	Promega	G8230
Software and algorithms		
GraphPad Prism 10	GraphPad Software, LLC	https://www.graphpad.com/
MassHunter Qualitative Analysis 10.0	Agilent Technologies	https://www.agilent.com/en
MassHunter Qualitative Analysis 10.1	Agilent Technologies	https://www.agilent.com/en

## Data Availability

All data reported in this paper will be shared by the lead contact upon request. This paper does not report original code. Any additional information required to reanalyze the data reported in this paper is available from the lead contact upon request.

## Highlights

- The mycobacterial ATP burst in the BacTiter-Glo assay is a lysis artifact
- Bead beating abolishes the ATP burst and enables more reliable ATP quantification
- Rapid bioluminescent screening for cell-wall-damaging compounds
- Detection of synergistic and antagonistic interactions with cell wall drugs
