# Supplementary material for: The mycobacterial ATP burst is a lysis artifact and serves as an assay for drug-induced cell wall damage
Source: Cell Rep. Author manuscript; Available in PMC 2026 Jun 16. (PMC13270088; doi:10.1016/j.celrep.2026.117286)
Supplement: 1 [file NIHMS2180937-supplement-1.pdf]

Cell Reports, Volume *45*

Supplemental information

The mycobacterial ATP burst is a lysis artifact and serves as an assay for drug-induced cell wall damage

Claire V. Mulholland, Bei Shi Lee, Alexander Chong, Jinhua Cui, Kevin Pethe, and Michael Berney

Table of contents

Supplementary Figures

S1. The isoniazid-induced ATP burst is not observed in heat-inactivated cultures.
S2. The ATP burst is an experimental artifact.
S3. Quantification of ATP by LC-MS.
S4. MmpL3 and DprE1 inhibitors induce an artefactual ATP burst in *Mtb*.
S5. Optimization of ATP screening for identification of cell wall inhibitors.
S6. Cross-resistance of *Mtb* to isoniazid and nialamide.
S7. Effect of LOPAC hits on the growth of virulent *Mtb* H37Rv.
S8. Effect of co-treatment of *Mtb* with isoniazid and bioenergetics inhibitors on ATP and survival.
S9. *Mtb* growth optimization in 96-well plate format.
S10. PDIM-positive *Mtb* shows an artifactual ATP burst with isoniazid.
S11. Whole-cell ATP dose-response of PDIM-positive *Mtb* to tetrahydrolipstatin and vancomycin.

Supplementary Tables

S1. MIC_{90} values and concentrations of compounds used in the isoniazid co-treatment assay.
S2. FICI assays for tetrahydrolipstatin and linezolid with isoniazid.

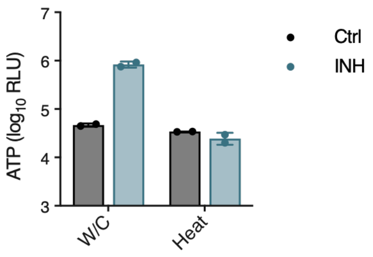

Figure S1. The isoniazid-induced ATP burst is not observed in heat-inactivated cultures. In a preliminary experiment, *Mtb* mc^{2}6230 at OD_{600} 0.33 was treated with isoniazid (INH, 5 µg/ml) for 24 h, and ATP was measured by the BacTiter-Glo™ assay using either whole-cell cultures (‘W/C’) or cultures inactivated by boiling at 100 °C for 30 minutes (‘Heat’). Mean ± SD, *n* = 2 technical replicates.

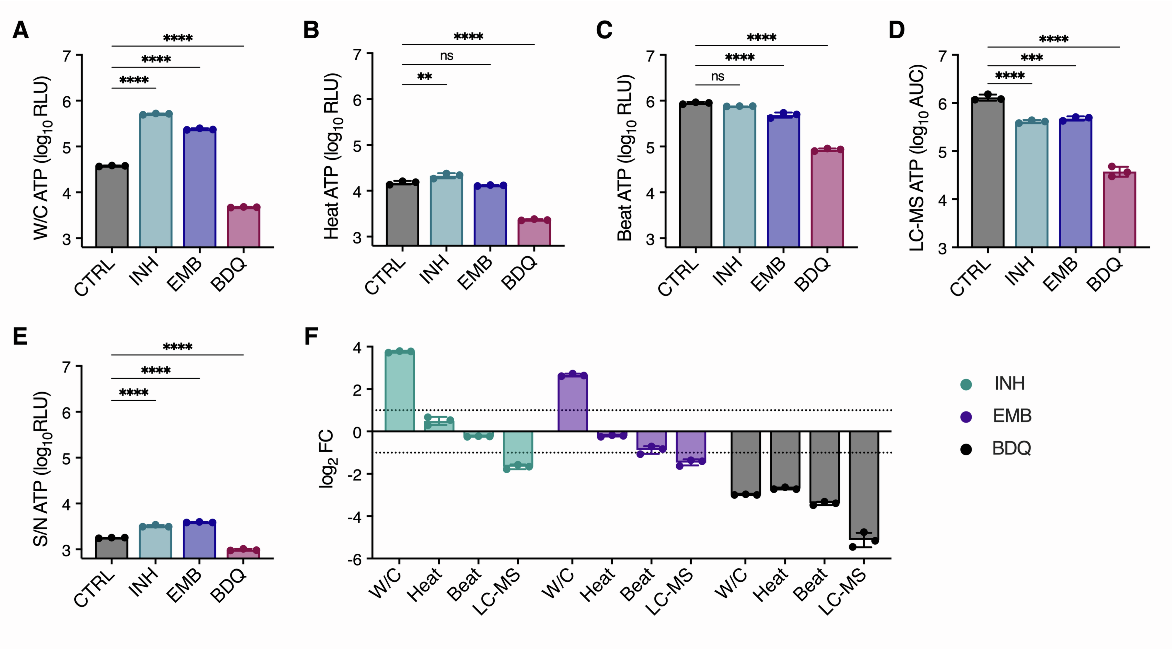

Figure S2. The ATP burst is an experimental artifact.
Mtb mc^{2}6230 at OD_{600} 0.33 was treated with isoniazid (INH, 3 µg/ml), ethambutol (EMB, 50 µg/ml), and bedaquiline (BDQ, 12.5 µg/ml) for 24 h. (A–C) ATP was measured using the BacTiter-Glo™ assay from either whole-cell cultures ('W/C') (A), the supernatant from cells lysed by heat inactivation ('Heat') (B), or supernatant from cells lysed by bead beating ('Beat') (C). (D) ATP levels in the same cultures measured by LC-MS. (E) ATP in the culture supernatant measured using the BacTiter-Glo™ assay. (F) Fold-change in ATP levels compared to untreated controls for the different methods shown in (A–D). The dotted lines indicate a 2-fold change. Mean ± SD, n = 3 replicate cultures. **$p < 0.01$, ***$p < 0.001$, ****$p < 0.0001$ by one-way ANOVA with Šídák's multiple comparison test (MCT). These data represent an independent repeat of the experiment shown in Figure 1.

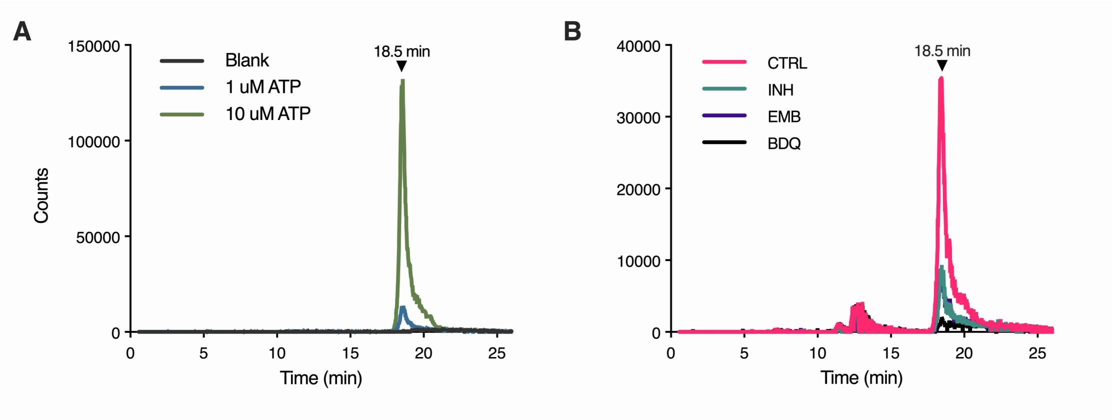

Figure S3. Quantification of ATP by LC-MS.

Extracted ion chromatograms for ATP ($[M–H]^-$, *m/z* = 505.9885) for ATP compound standards (A) and sample extracts (B). One representative extract from each condition is shown.

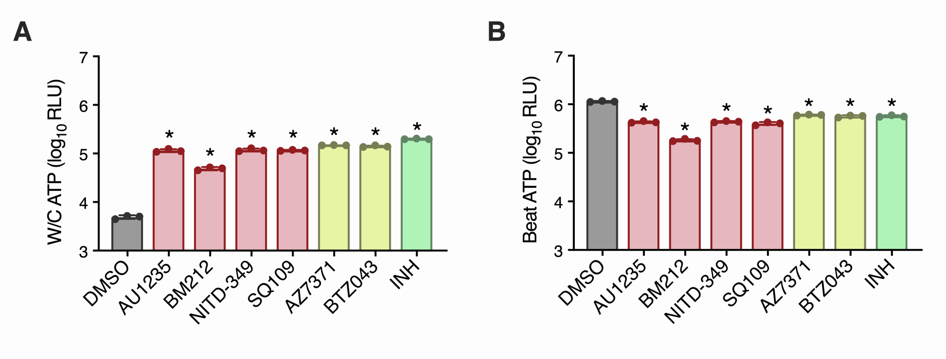

Figure S4. MmpL3 and DprE1 inhibitors induce an artefactual ATP burst in *Mtb*.
Mtb mc[2]6230 at OD_{600} 0.033 was treated for 24 h with compounds at 10 × MIC_{90} and ATP was measured using the BacTiter-Glo™ assay from either whole-cell cultures (A) or cells lysed by bead beating (B). MmpL3 inhibitors are shown in red and DprE1 inhibitors in yellow. INH = isoniazid. Mean ± SD, n = 3 replicate cultures. *$p < 0.0001$ compared to the DMSO control by one-way ANOVA with Dunnett's MCT. This experiment was performed once.

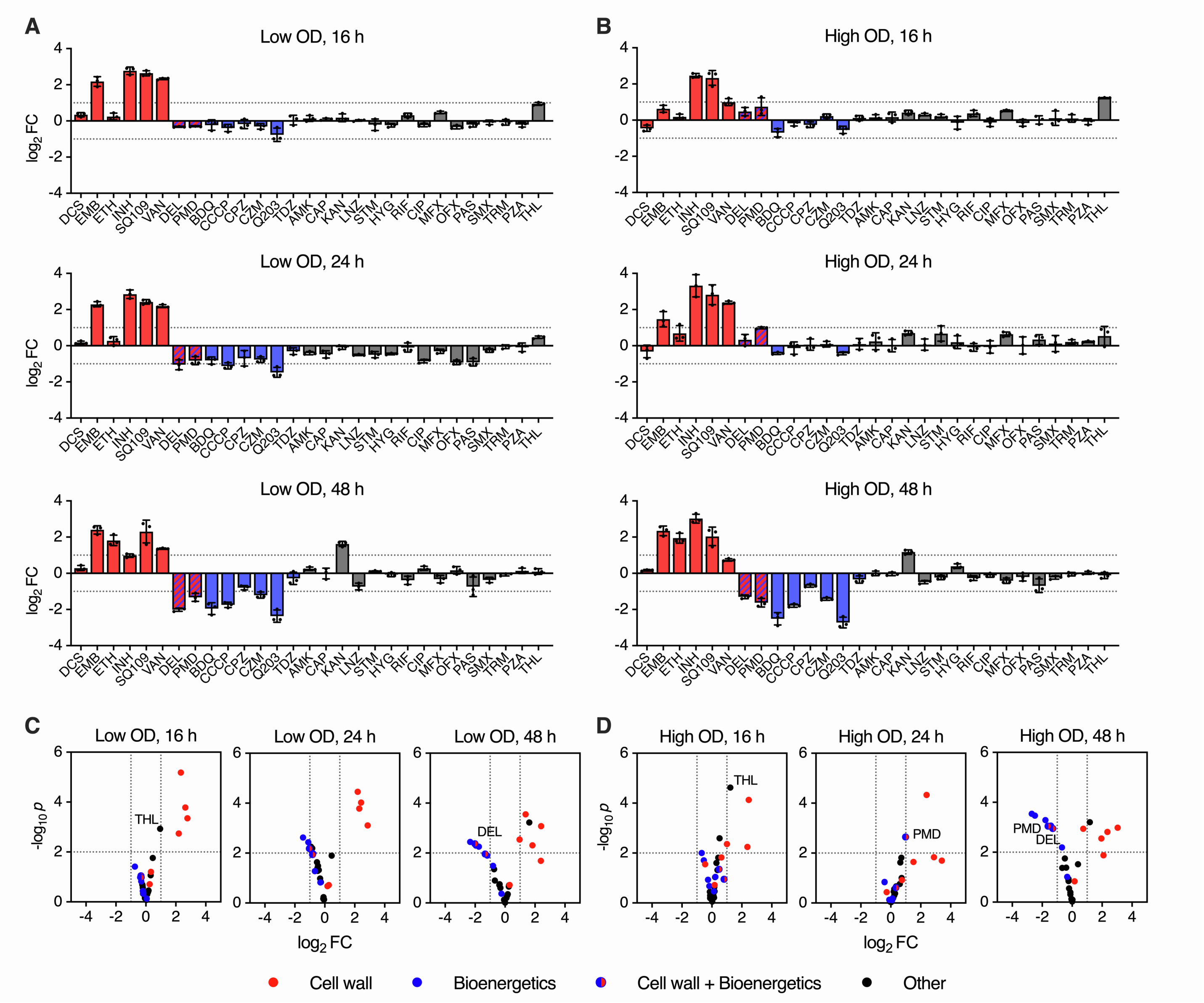

Figure S5. Optimization of ATP screening for identification of cell wall inhibitors.
(A–B) *Mtb* mc^{2}6230 was diluted to OD_{600} 0.033 ('low OD') (A) or OD_{600} 0.33 ('high OD') (B) and treated with 28 reference compounds (see Table 1) at 10 µM alongside DMSO-only controls. Cultures were incubated in 96-well microtiter plates for 16, 24, or 48 h, and ATP was measured using the BacTiter-Glo™ whole-cell assay. Log_2 fold-changes relative to DMSO-only controls are shown. Mean ± SD, n = 3 replicate wells. (C) Volcano plots showing responses relative to DMSO-only controls for the low-density cultures from (A), and (D) the high-density cultures from (B). Dotted lines indicate a 2-fold change and p-value of 0.01. See also Figure 3A–B, which represents an independent repeat of the low OD, 24 h experiment.

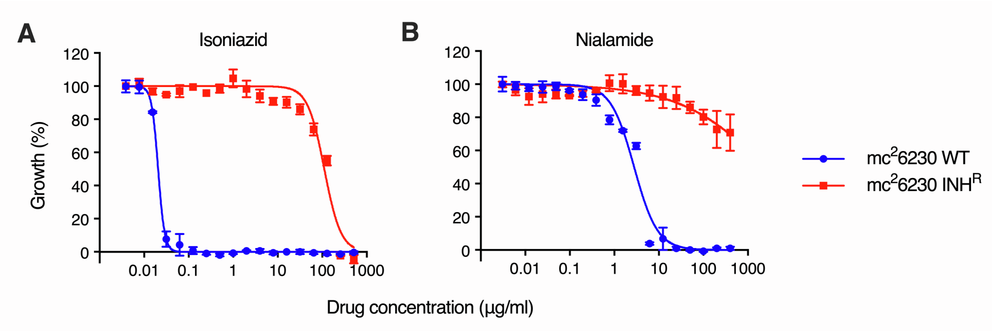

Figure S6. Cross-resistance of *Mtb* to isoniazid and nialamide.
Minimum inhibitory concentration (MIC) assays of *Mtb* mc^{2}6230 wildtype (WT) and an isoniazid-resistant mutant (INH^R) with isoniazid (A) and nialamide (B). Growth was measured by OD_{600} and normalized to no-drug control wells. The isoniazid-resistant mutant has a *katG* 1627AG>A frameshift mutation, rendering it unable to activate isoniazid. Mean ± SD, $n = 2$ replicate wells.

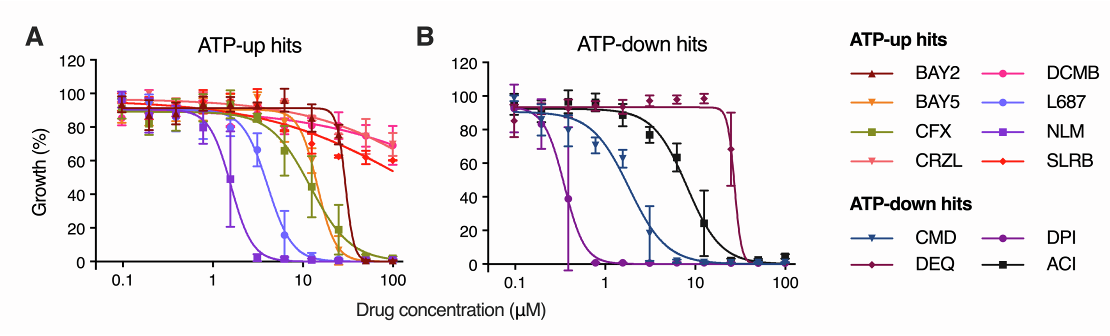

Figure S7. Effect of LOPAC hits on the growth of virulent *Mtb* H37Rv.

MIC assays for PDIM-positive *Mtb* H37Rv AE1001 with selected LOPAC compounds that either significantly increased the ATP signal by >2-fold (‘ATP-up’ hits) (A) or significantly decreased the ATP signal by >80% (‘ATP-down’ hits) (B) (see Table 2). The culture media was additionally supplemented with 0.1 mM propionate to support PDIM production. Growth was measured by OD_{600} and normalized to no-drug control wells. Mean ± SD, *n* = 4 replicate wells from two independent experiments.

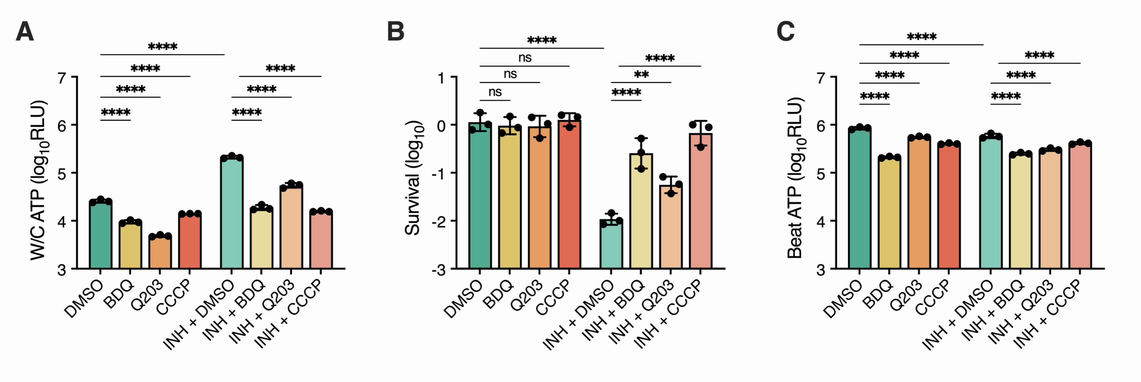

Figure S8. Effect of co-treatment of *Mtb* with isoniazid and bioenergetics inhibitors on ATP and survival.

Mtb mc^{2}6230 at OD_{600} 0.33 was treated with isoniazid (INH, 0.1 µg/ml), bedaquiline (BDQ, 2.5 µg/ml), Q203 (200 nM), and CCCP (20 µg/ml) for 24 h. (A) ATP measured using the BacTiter-Glo™ whole-cell assay. (B) Relative survival following drug treatment. Cultures were plated for CFU before and after treatment to assess viability. The starting cell density was 2×10^8 CFU/ml. (C) ATP measured using the BacTiter-Glo™ beat assay. Mean ± SD, $n = 3$ replicate cultures. **$p < 0.01$, ****$p < 0.0001$ by one-way ANOVA with Šídák's MCT. This experiment was repeated with similar results.

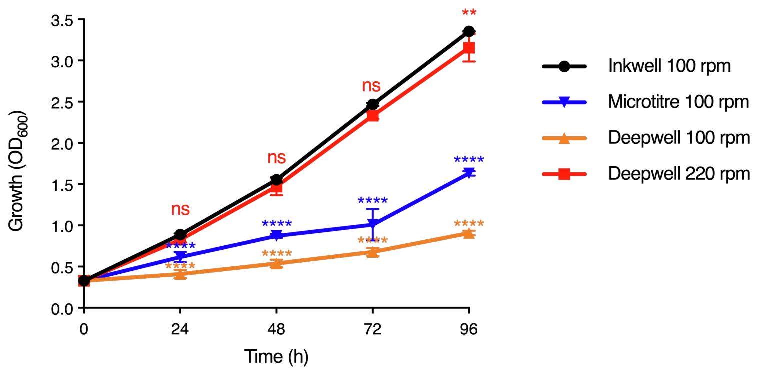

Figure S9. *Mtb* growth optimization in 96-well plate format.
Mtb mc^{2}6230 cultures at OD 0.33 were grown in either inkwell bottles, a standard 96-well microtiter plate, or 2 ml V-bottom 96-deepwell blocks, shaken gently at 100 rpm or vigorously at 220 rpm. The culture volume was 5 ml for inkwells and 200 µl for 96-well plates and deepwell blocks. Deepwell blocks were sealed with sterile foil seals, contained in sealed bags and secured using the Khuner Duetz System (Basel, Switzerland) for fast shaking. Samples from both inkwells and 96-well plates were diluted in formalin and measured in cuvettes using a standard spectrophotometer. Mean ± SD, n = 3 replicate inkwell cultures, and n = 3 samples from replicate wells for 96-well plates and deepwell blocks. **$p < 0.01$, ****$p < 0.0001$ compared to inkwell cultures by two-way ANOVA with Dunnett's MCT. Deepwell blocks shaken at 220 rpm reproducibly resulted in growth comparable to inkwells.

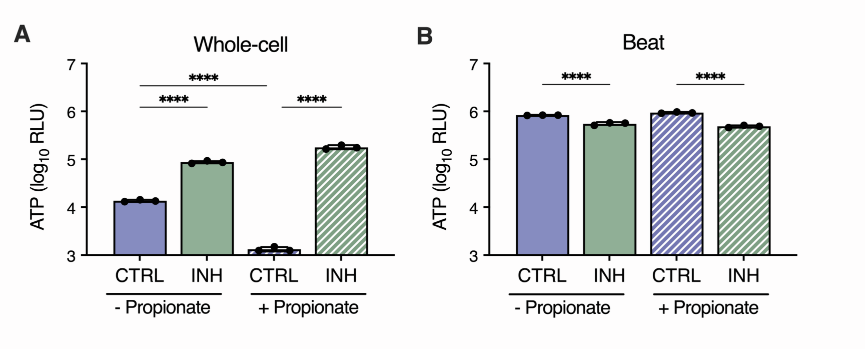

Figure S10. PDIM-positive *Mtb* shows an artifactual ATP burst with isoniazid.
PDIM-positive *Mtb* $mc^2$6230 AE1601 was diluted to OD_{600} of 0.05 into 7H9/OADC/glycerol/ tyloxapol media with and without 0.1 mM propionate and treated with isoniazid (INH, 0.07 µg/ml) for 24 h. ATP was measured using the BacTiter-Glo™ whole-cell (A) and beat assays (B). Mean ± SD, $n = 3$ replicate cultures. ****$p < 0.0001$ by one-way ANOVA with Šídák's MCT.

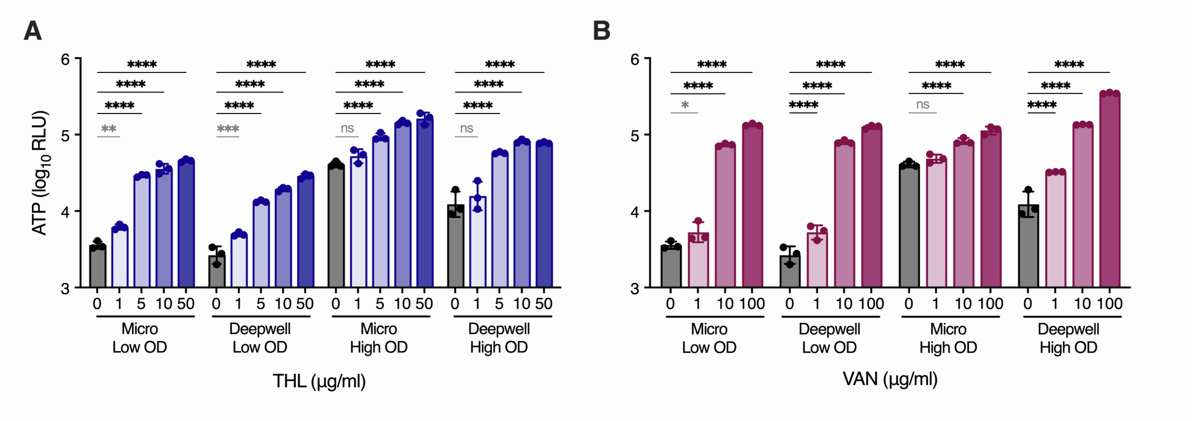

Figure S11. Whole-cell ATP dose-response of PDIM-positive *Mtb* to tetrahydrolipstatin and vancomycin.

PDIM-positive *Mtb* mc^{2}6230 AE1601 was diluted to either OD 0.033 ('low OD') or OD 0.33 ('high OD') and treated with different concentrations of tetrahydrolipstatin (A) or vancomycin (B) for 24 h, and ATP was measured using the BacTiter-Glo™ whole-cell assay. Cultures were treated in either standard 96-well microtiter plates with gentle shaking at 100 rpm or in 96-deepwell blocks with vigorous shaking at 220 rpm (see Figure S9). Mean ± SD, n = 3 replicate wells. *$p < 0.05$, **$p < 0.01$, ***$p < 0.001$, ****$p < 0.0001$ by two-way ANOVA with Dunnett's MCT. Comparisons with ≤2-fold changes are indicated in grey and >2-fold in black. Based on these results, we opted to use low-density cultures with 1 µg/ml of each drug in 96-well microtiter plates for the cotreatment assay in Figure 5E, as this induced minimal response compared to DMSO-only (≤2-fold), while strong responses were elicited at higher concentrations.

Supplementary Tables

Table S1. MIC_{90} values and concentrations of compounds used in the isoniazid co-treatment assay.

Concentrations are reported in μg/ml, except for Q203, which is in nM. MICs were measured in *Mtb* $mc^2$6230 using the microbroth dilution method.

Abbr.	Name	MIC_{90}	conc1	conc2
INH	Isoniazid	0.05	N/A	N/A
AMK	Amikacin	0.5	2	5
BDQ	Bedaquiline	0.25	0.5	1.25
CCCP	Carbonyl cyanide 3-chlorophenylhydrazone	10	20	50
CZM	Clofazimine	0.5	0.5	1.25
LNZ	Linezolid	1	2	5
MFX	Moxifloxacin	0.1	0.2	0.5
PAS	4-Aminosalicylic acid	0.1	0.2	0.5
Q203	Q203	20 (nM)	40 (nM)	100 (nM)
RIF	Rifampicin	0.05	0.1	0.25
STM	Streptomycin	1	4	10
THL	Tetrahydrolipstatin	16 – 32	50	125

Table S2. FICI assays for tetrahydrolipstatin and linezolid with isoniazid.

Data are shown as growth normalized to no-drug control wells. Yellow wells indicate the MIC_{90} for each drug alone. Wells nearest half the MIC for each drug (orange wells) were used to calculate the FIC index as follows: FICI = $(MIC_{AB}/MIC_A) + (MIC_{BA}/MIC_B)$. Data show mean growth from two replicate plates.

		**INH** (µg/ml)									
		**0.4**	**0.2**	**0.1**	**0.05**	**0.025**	**0.0125**	**0.0063**	**0.0031**	**0.0016**	**0**
**THL** (µg/ml)	**32**	0.0%	-3.9%	1.2%	-0.8%	1.2%	1.2%	1.6%	1.7%	1.8%	0.0%
	**16**	1.0%	0.3%	1.5%	0.7%	1.4%	2.4%	3.0%	3.6%	3.4%	2.6%
	**8**	0.1%	0.1%	-0.7%	0.0%	0.7%	4.8%	13.7%	12.6%	11.8%	13.4%
	**4**	-1.2%	-0.1%	-0.7%	-2.7%	-0.3%	13.6%	25.8%	36.3%	37.3%	40.2%
	**2**	-0.3%	-1.5%	-0.4%	-1.1%	-0.7%	39.6%	44.3%	46.9%	47.2%	48.2%
	**0**	-1.3%	-2.0%	-0.3%	-1.1%	60.2%	92.1%	94.6%	98.5%	102.0%	97.8%

		**INH** (µg/ml)									
		**0.4**	**0.2**	**0.1**	**0.05**	**0.025**	**0.0125**	**0.0063**	**0.0031**	**0.0016**	**0**
**LNZ** (µg/ml)	**1**	0.0%	-0.4%	0.4%	0.9%	1.1%	2.0%	6.1%	8.0%	3.6%	4.3%
	**0.5**	0.1%	0.3%	2.0%	1.3%	1.9%	54.7%	66.9%	72.8%	52.4%	67.8%
	**0.3**	-0.5%	0.5%	-0.3%	0.6%	34.4%	85.5%	88.5%	89.7%	81.8%	94.4%
	**0.1**	-0.5%	0.1%	1.0%	-0.8%	70.8%	101.4%	99.7%	100.7%	96.9%	107.8%
	**0.1**	-0.2%	-0.2%	0.7%	0.8%	72.8%	100.2%	101.2%	103.7%	103.8%	107.4%
	**0**	-1.1%	-1.4%	-0.6%	-0.7%	67.6%	92.6%	102.2%	103.8%	102.2%	106.3%
